# Impact of body mass index on outcome in patients undergoing coronary artery bypass grafting and/or valve replacement surgery

**DOI:** 10.5935/1678-9741.20150027

**Published:** 2015

**Authors:** Vinícius Eduardo Araújo Costa, Silvia Marinho Ferolla, Tâmara Oliveira dos Reis, Renato Rocha Rabello, Eduardo Augusto Victor Rocha, Célia Maria Ferreira Couto, José Carlos Ferreira Couto, Alduir Bento

**Affiliations:** 1 Hospital Vera Cruz (HVC), Belo Horizonte, MG, Brazil.

**Keywords:** Body mass index, Obesity, Thoracic surgery, Myocardial revascularization, Mitral valve prolapse

## Abstract

**Objective:**

This study aimed to analyze the impact of body mass index on outcomes of 101 patients undergoing coronary artery bypass grafting, valve replacement, or combined valve/ coronary artery bypass grafting surgery in a private hospital in Belo Horizonte, Brazil.

**Methods:**

This was a prospective cross-sectional study of patients undergoing cardiac surgery from May 2009 to December 2012. All patients were followed up from the first day of admission until discharge or death. Patients were divided into three groups according to BMI: normal weight, overweight, and obese. The main outcome measure was the association between BMI and postoperative morbidities and mortality.

**Results:**

Multivariate analysis identified obesity as an independent predictor of increased risk of surgical reintervention (odds ratio [OR] 13.6; 95%CI 1.1 - 162.9; *P*=0.046) and reduced risk of bleeding (OR 0.05; 95% CI 0.09 - 0.69; *P*=0.025). Univariate analysis showed that obesity was associated with increased frequency of wound dehiscence (*P*=0.021). There was no association between BMI and other complications or mortality in univariate analysis. There was also no association between body mass index and duration of cardiopulmonary bypass, aortic clamping, mechanical ventilation, and intensive care unit or hospital stay.

**Conclusion:**

Obese individuals undergoing coronary artery bypass grafting, valve replacement, or combined surgery have a higher postoperative risk of surgical reintervention and lower chances of bleeding.

## Abstract (pt)

**Objetivo:**

Analisar o impacto do índice de massa corporal no desfecho de 101 pacientes submetidos à cirurgia revascularização do miocárdio, troca valvar ou cirurgia cardíaca combinada em um hospital privado de Belo Horizonte, Minas Gerais.

**Métodos:**

Trata-se de um estudo transversal com inclusão prospectiva de pacientes submetidos à cirurgia cardíaca no período de maio de 2009 a dezembro de 2012. Todos os pacientes foram acompanhados do primeiro dia de internação até a alta hospitalar ou óbito. Os pacientes foram divididos em três grupos definidos pelo índice de massa corporal aferido no pré-operatório: eutrófico, sobrepeso e obeso. O principal desfecho avaliado neste estudo foi a associação entre índice de massa corporal e morbimortalidade pós-operatória.

**Resultados:**

análise multivariada revelou obesidade como preditor independente de aumento nas chances de reintervenção cirúrgica por deiscência de sutura de esterno (OR 13,6; IC95% 1,1-162,9; *P*=0,046) e redução no risco de sangramento (OR 0,05; IC95% 0,09-0,69; *P*=0,025). Na análise univariada, obesidade também foi associada a maior frequência de deiscência de sutura (*P*=0,021). Estado nutricional não foi associado à presença de outras complicações no pós-operatório e nem de mortalidade, ainda na análise univariada. No intraoperatório não houve diferença nos tempos de circulação extracorpórea e de pinçamento aórtico. No pós-operatório, os tempos de ventilação mecânica e de internação na unidade de terapia intensiva ou hospital foram semelhantes entre os pacientes eutróficos, com sobrepeso e obesos.

**Conclusão:**

Pacientes obesos apresentam risco aumentado de reintervenção cirúrgica por deiscência de sutura e menor risco sangramento no pós-operatório de cirurgia de revascularização do miocárdio, troca valvar ou cirurgia cardíaca combinada.

**Table:** 

**Abbreviations, acronyms & symbols**	
BMI	Body mass index
CABG	Coronary artery bypass grafting
CAD	Coronary artery disease
CPB	Cardiopulmonary bypass
ICU	Intensive care unit
MV	Mechanical ventilation

## INTRODUCTION

The prevalence of obesity has been increasing in Brazil and developed countries^[1,2]^. Research by the Brazilian Institute of Geography and Statistics^[1]^ has shown that almost half of Brazilians (48%) have excess weight. It is well known that obesity increases the risk of coronary artery disease (CAD)^[3]^ and is associated with increased mortality in this population as well as in the general population^[4,5]^. In 2009, a total of 209,029 Brazilian patients were hospitalized due to CAD, with a mortality rate of 6.04%^[5]^.

Given the endemic nature of obesity in the contemporary world, numerous patients with excess body weight are expected to require cardiac surgery^[6-8]^. Despite advances in clinical treatment and percutaneous procedures, coronary artery bypass grafting (CABG) is still considered a safe surgical method even in at-risk populations and is widely performed in Brazil and worldwide for treatment of obstructive CAD^[9-14]^.

Conflicting data has been published on the influence of obesity on morbidity and mortality in cardiac surgery. Studies in different countries have documented an "obesity paradox", suggesting a neutral or beneficial effect of excess weight on the outcome of patients undergoing coronary angioplasty, surgery for valve replacement, and CABG^[15-20]^. In a retrospective Brazilian study of 290 elderly patients who underwent CABG, obesity had a protective association with pulmonary dysfunction, risk of readmission, and mortality, although it was a risk factor for renal dysfunction during the postoperative period^[21]^. However, some authors have identified obesity, diabetes, and chronic obstructive pulmonary disease as independent risk factors of mediastinitis after cardiac surgery^[22]^.

Data on the adverse or protective effects of body weight in Brazilian patients undergoing cardiac surgery remain scarce. The current study was designed to assess the preoperative impact of BMI on morbidity and mortality in the postoperative period in adult and elderly patients undergoing CABG, valve replacement, or combined cardiac surgery.

## METHODS

### Study Design and Sample

This was a prospective cross-sectional study conducted between May 2009 and December 2012 in a private hospital in Belo Horizonte, Minas Gerais, Brazil, with a residency training program in cardiovascular surgery. In all, 101 of 118 patients undergoing elective CABG or valve replacement surgeries were assessed. The same team performed all surgical procedures. Adult and elderly patients (aged 60 years or over) were included. Patients younger than 18 years and those undergoing other surgical modalities such as excision of atrial myxoma and Bentall - De Bono surgery were excluded. All patients were followed up from the first day of hospitalization (pre-operative) until hospital discharge or death (when this occurred during hospitalization).

### Ethical Aspects

The study participants were part of a wide-ranging study, entitled, "The use of nutritional assessment as a predictor of risk of complications in patients undergoing cardiac surgery", which was approved by the Research Ethics Committee of Vera Cruz Hospital, Belo Horizonte , Minas Gerais, under number 097/09. All participants voluntarily signed an informed consent form.

### Data Collection and Definitions

Data were collected prospectively. The clinical variables included in the study were divided into pre-operative, peri-operative, and postoperative variables, as shown in Table 1.

**Table 1:** Clinical variables in the study.

Preoperative variables	Perioperative variables	Postoperative variables
Age	Surgical procedure	APACHE II Score
Gender	CPB time (min)	Infectious complications
BMI (kg/m^2^)	Aorta clamping time (min)	Cardiovascular complications
Albumin (g/dL; normal ≥3.5g/dL)		Surgical reintervention
Smoking (current)		Acute kidney injury
Previous heart surgery		Minor complications
Heart failure (LVEF < 45%)		Mortality (up to 30 DAS)
CKD (Cr ≥2.5mg/dL or dialysis)		MV duration (days)
COPD (drug therapy)		ICU stay (days)
Glucose intolerance/DM (plasma glucose ≥ 100mg/dL/ drug therapy)		Total lenght of hospital stay (days)
HBP (≥130/85 mmHg/ drug therapy)		
Dyslipidemia (HDL<40mg/dL in men and <50mg/dL in women and/or TG ≥150mg/dL/ drug therapy)		

CPB=cardiopulmonary bypass; DM=diabetes mellitus; DAS= days after surgery; COPD=chronic obstructive pulmonary disease; APACHE II score=Acute Physiology and Chronic Health Evalution; HBP=high blood pressure; LVEF=left ventricle ejection fraction; HDL=high density lipoprotein cholesterol; BMI=body mass index CKD=chronickidney disease; TG=triglycerides; ICU=intensive care unit;MV=mechanicalventilation

Clinical and demographic data including the presence of associated comorbidities and risk factors were obtained from medical history collected during the pre-operative evaluation. Nutritional parameters including body mass index (BMI) and albumin concentration were measured during the pre-operative period. The BMI was calculated using the Quetelet's index^[23]^. BMI was classified according to criteria from the World Health Organization (WHO)^[24]^. The study population was divided into three groups: normal weight (BMI between 18.5 and 24.9 kg/m^2^), overweight (BMI ≥ 25 kg/m^2^ and < 30 kg/m^2^), and obese (BMI ≥ 30 kg/m^2^).

All surgical procedures were performed under balanced intravenous general anesthesia. Median sternotomy was performed in all patients. After systemic heparinization, cardiopulmonary bypass was instituted between the ascending aorta and the right atrium using a 2-stage cannula or cannulation of both venae cavae. Cardiac protection was instituted by means of intermittent clamping of the aorta and crystalloid cardioplegia with blood dilution during CABG and valve replacement surgery, respectively. The duration of cardiopulmonary bypass (CPB) and aorta clamping were measured during the perioperative period.

The Acute Physiology and Chronic Health Evaluation (APACHE II) score was calculated on patient admission to the intensive care unit (ICU) during the immediate post-operative period, and the duration of mechanical ventilation (MV), time of stay in the ICU, total length of hospital stay, complications, and postoperative mortality (when this occurred up to 30 days after the surgical procedure) were also recorded.

Complications were categorized as: 1) infectious; 2) cardiovascular; 3) requiring surgical reintervention for sternal wound dehiscence; 4) increased bleeding; 5) acute kidney injury; and 6) minor complications. Infectious complications were defined as pneumonia, urinary tract infection, sepsis, septic shock, mediastinitis, infections of the lower limbs, or endocarditis. Cardiovascular complications were defined as acute myocardial infarction, cardiogenic shock, atrial fibrillation, stroke, heart failure, transient ischemic attack, or lower limb ischemia. Increased bleeding was characterized as the need for blood transfusion or surgical reintervention. Acute kidney injury in the postoperative period was defined as serum creatinine levels greater than or equal to 2.0 or requiring hemodialysis. The following were considered minor complications: pericardiotomy syndrome, pleural effusion, pressure ulcers, lowers limb wounds, and sinusitis.

The main outcome assessed in this study was the association between BMI and complications during the CABG or valve replacement postoperative period.

### Statistical Analysis

Descriptive analysis of the data was performed; proportions were calculated for categorical variables and minimum, median, maximum, average and standard deviation were calculated for continuous variables. Chi-square and Kruskal-Wallis tests were used to assess independence between groups and for comparison of medians, respectively^[25]^.

The multivariate analysis used an adjusted multinomial regression model that considered the overweight group as the reference. The model was adjusted for postoperative complications; those without significant *P* values were retained due to clinical significance^[25]^.

Analyses were performed using STATA version 12.0 (Stata Corporation, College Station, Texas), with a 5% significance level.

## RESULTS

**Fig. 1:**
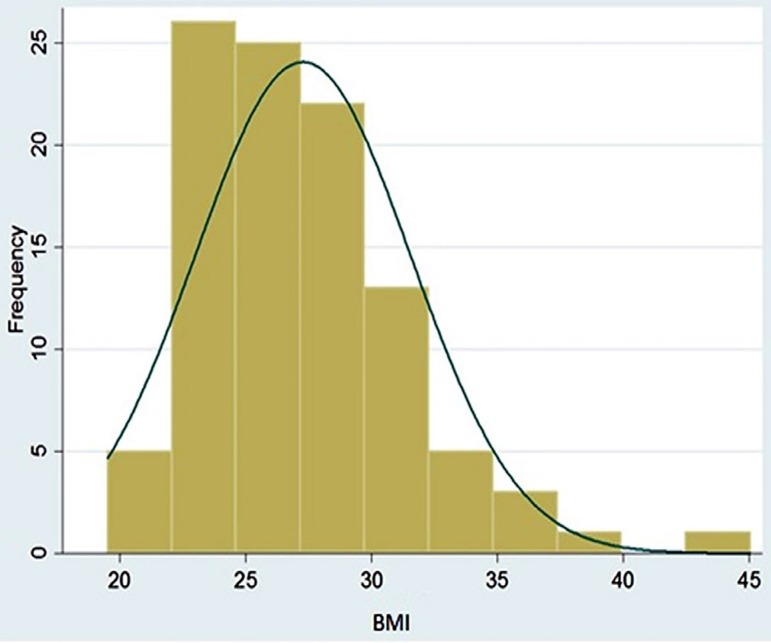
Body mass index distribution in pre-operative patients for heart surgery.

A total of 101 patients were included in this study and most were male (73.3%). Sixty-one percent were elderly, with a mean age of 61.8±10.1 years. The mean BMI was 27.3±4.3 kg/m^2^ (Figure 1). Approximately 32.0% of patients had a healthy weight, 47.5% were overweight, and 20.8% were obese. The mean serum albumin level in the preoperative period was 4.1±0.6 g/dL. None of the participants was malnourished in the pre-operative period according to BMI classification. A minority (9.0%) had serum albumin less than 3.5 g/dL. When the study population was stratified according to BMI classification, there was no significant difference in the frequency of elderly individuals between the groups (Table 2).

**Table 2:** Demographic characteristics, comorbidities, and risk factors during preoperative evaluation of patients undergoing heart surgery.

		BMI		
Variable	Normal weight	Overweight	Obesity	*P* value
	n=32	n=48	n=21	
	%	%	%	
Male gender	31.1%	47.3%	21.6%	0.939
Elderly	32.3%	50.0%	17.7%	0.624
GI/ DM	37.7%	37.7%	24.6%	0.015*
HF	55.6%	33.3%	11.1%	0.267
HBP	26.2%	48.8%	25.0%	0.010*
COPD	75.0%	25.0%	0.0%	0.151
CKD	0.0%	100.0%	0.0%	0.100
Dyslipidemia	31.2%	45.5%	23.4%	0.507
Smoking	47.1%	29.4%	23.5%	0.222
PHS	40.0%	60%	0.0%	0.099

BMI=body mass index; CKD=chronic kidney disease; COPD=chronic obstructive pulmonary disease; DM=diabetes mellitus; HBP=high blood pressure; GI=glucose intolerance; HF=heart failure; PHS=previous heart surgery

Analysis of comorbidities and risk factors identified in the pre-operative period for the entire sample population revealed that the majority of patients were hypertensive (83.2%), dyslipidemic (76.2%), and glucose intolerant or diabetic (68.3%). Approximately 15% had previous history of cardiac surgery and aroundy 17% were smokers. When classified by BMI, the only comorbidities and/or risk factors that showed statistical differences between the groups were diabetes and hypertension that were more prevalent among overweight individuals (Table 2).

CABG was the most common surgical procedure in this population (71% of cases), followed by valve replacement (23%) and combined surgery (6%). There was no difference in the frequency of CABG or valve replacement between the normal, overweight, and obese groups (*P*=0.241).

On average, patients remained in CPB for 79.3±24.9 minutes in the peri-operative period, and the average aortic clamping time was 37.6±16.9 minutes. The median CPB times in healthy, overweight, and obese individuals were 73 (interquartile range [IQR]: 58-97 min), 80.5 (IQR: 67-93 min), and 71 min (IQR: 62-81 min), respectively. The median aorta clamping times were 35 (IQR: 28-52 min), 37.5 (IQR: 29-43 min), and 31 min (IQR: 25-35min), respectively, for the same patient groups. There were no significant differences in median CPB time (*P*=0.215) and aortic clamping (*P*=0.064) between groups stratified by BMI.

In the immediate postoperative period, 44% of patients had APACHE II scores higher than 8.0. This score was also not different among normal BMI, overweight, and obese patients (*P*=0.458).

The median post-surgical ICU and MV durations were 5 days (min: 3; max: 102) and 1 day (min: 1; max: 35), respectively. The median hospital stay was 16 days (min: 4; max: 117). There was no difference between MV, ICU, and hospitalization duration between patient groups (Table 3). Most patients were discharged from hospital (94.1%), while postoperative mortality occurred in 6 of 101 patients (5.9%).

**Table 3:** Comparison of MV, ICU, and hospital stay postoperative times from patients undergoing heart surgery.

					BMI					
Days		Normal weight			Overweight			Obesity		
	n	Median	Q1-Q3	n	Median	Q1-Q3	n	Median	Q1-Q3	*P* value
MV	32	1	1-2.5	48	1	1-2	21	1	1-2	0.578
ICU	32	5	4-9	48	5	3-6.5	21	5	4-6	0.741
Hospital stay	32	17	10-23	48	16	10-26	21	13	9-21	0.694

BMI=body mass index; CABG=coronary artery bypass grafting; MV=mechanical ventilation

Slightly more than half of the patients (50.5%) had no postoperative complications. The univariate analysis revealed an association between obesity and surgical reintervention due to sternal wound dehiscence (*P*=0.021). All patients with sternal wound dehiscence were obese (Table 4). Univariate analysis revealed no association between BMI and postoperative mortality (*P*=0.15).

**Table 4:** Association between BMI and postoperative complications from CABG and/or valve replacement (univariate analysis).

		BMI		
Variable	Normal	Overweight	Obesity	*P* value
	%	%	%	
Cardiovascular complications	32.1	46.2	21.8	0.854
Infectious complications	28.4	48.7	23.0	0.436
Sternal wound dehiscence	0.0	0.0	10.0	0.021*
Bleeding	32.4	43.2	24.3	0.249
Acute kidney failure	31.5	47.8	20.7	0.981
Minor complications	32.1	45.2	22.6	0.501

BMI=body mass index; CABG=coronary artery bypass grafting

The multivariate analysis was adjusted for classes of complications (cardiovascular, infectious, requirement for surgical reintervention due to sternal wound dehiscence, bleeding, acute kidney injury, and minor complications) using the overweight group as a reference. Obesity was an independent predictor for surgical reintervention due to sternal wound dehiscence (odds ratio [OR]: 13.6; 95% confidence interval [95%CI]: 1.1-162.9; *P*=0.046) and a protective factor for bleeding (OR: 0.05; 95%CI: 0.09-0.69; *P*=0.025), as shown in Table 5. Mortality was not entered in the multivariate model due to the low number of deaths.

**Table 5:** Obesity and postoperative complications risk at CABG and/or valve replacement (multivariate analysis).

			BMI					
Variable		Normal weight					Obesity	
	OR	95% IC	*P* value			OR	95% IC	*P* value
Cardiovascular complications	0.83	0.22-3.13	0.784			0.83	0.22-3.13	0.784
Infectious complications	2.27	0.65-7.98	0.200			2.27	0.65-7.98	0.200
Sternal wound dehiscence	2.18	0.42-11.43	0.357			2.18	0.42-11.43	0.357
Bleeding	0.37	0.08-1.65	0.194			0.37	0.08-1.65	0.194
Acute kidney injury	0.74	0.11-5.18	0.761			0.74	0.11-5.18	0.761
Minor complications	0.73	0.18-3.01	0.661			0.73	0.18-3.01	0.661

BMI=body mass index; CABG=coronary artery bypass grafting

## DISCUSSION

To our knowledge, this was the first prospective Brazilian study designed to examine the impact of BMI on the outcome of patients undergoing CABG or valve replacement. The main finding of this study was that obesity in the preoperative period could be considered a predictor of risk for surgical reintervention by wound dehiscence and may reduce the risk of bleeding in the postoperative period of cardiac surgery. Although obesity appears to increase the frequency and risk of sternal wound dehiscence, it was not associated with other complications in the postoperative period or with increased mortality.

Univariate and multivariate analyses revealed an association between obesity and increased frequency or risk of reoperation for sternal wound dehiscence in this study. We believe that the increased subcutaneous tissue thickness in individuals with excessive body weight may contribute to this complication. In support of our findings, a cohort study published in 2014 involving 5,815 patients who underwent CABG also noted that obesity was a predictive factor for sternal wound dehiscence, along with diabetes and female sex^[26]^.

In multivariate analysis, obesity was a protective factor for increased bleeding in the CABG or valve replacement postoperative period. The observation that obesity is a protective factor for bleeding in the postoperative period of cardiac surgery was expected, since obese individuals have abundant mediastinal fat and large abdominal pressure, which leads to increased intrathoracic pressure that compresses sites of minor bleeding. In addition, reduced administered volume after CPB and less hemodilution in obese patients may also contribute to lower risk of postoperative bleeding. Thus, obese patients have significantly lower risks of surgical reintervention due to bleeding than non-obese or underweight patients^[27,28]^.

We found no association between obesity and the presence of infectious or cardiovascular complications, acute kidney injury, or minor complications. Gurm et al . analyzed data from 1,526 patients during the CABG postoperative period and also reported no difference in the incidence or risk of major complications (death, myocardial infarction, stroke), cardiopulmonary events (heart failure, cardiogenic shock, reintubation), and wound infection among obese patients^[29]^.

In the present study, there was no association between obesity and increased risk of mortality. However, two recent meta-analyses have noted that obese individuals have reduced risk of mortality after coronary revascularization^[18,30]^. In other studies, obese patients undergoing valve replacement surgery also showed superior survival time^[19,20]^.

Our results showed lower average durations of CPB and aortic clamping than those described by other authors (79.3±24.9 min *vs.* 103.4±35.1 min and 37.6±16.8 min *vs.* 74.8±24.2 min, respectively)^[19]^. In our population, BMI was not associated with changes in CPB duration and aortic clamping, possibly due to little intrathoracic anatomical variation in non-obese and obese patients. In addition, the duration of MV, ICU stay, and total length of hospital stay also did not differ according to BMI in the present study. These findings are consistent with data of other publications^[19,31-33]^.

This study has some methodological limitations, especially with regard to the sample size. It was not possible to evaluate the usefulness of BMI as a predictor of mortality in the multivariate analysis. However, this is perhaps the only prospective study that provides data about the influence of BMI outcomes of adult and elderly patients undergoing myocardial revascularization or prosthetic valve replacement in Brazil. In addition, our results contribute to knowledge about the obesity paradox in cardiovascular surgery.

## CONCLUSION

Obese patients undergoing CABG or valve replacement may be at increased risk of surgical reintervention by wound dehiscence and seem to be more protected from the risk of increased postoperative bleeding. Obesity does not appear to be related to increased incidence of other complications or postoperative mortality. However, larger studies are needed to establish definitive conclusions about the impact of obesity on mortality in the CABG and valve replacement postoperative period.

**Table:** 

**Authors’ roles & responsibilities**	
VEAC	Manuscript writing.
SMF	Analysis and/or interpretation of data; Statistical analysis; final approval of the manuscript; study design; implemen-tation of projects and/or experiments; manuscript writing or critical review of its content
TOR	Conduct of operations and/or experiments
RRR	Final approval of the manuscript; study design
EAVR	Final approval of the manuscript; manuscript writing or crit-ical review of its content
CMFC	Conception and design; implementation of projects and/or experiments
JCFC	Conception and design; implementation of projects and/ or experiments; manuscript writing or critical review of its content
AB	Analysis and/or interpretation of data; statistical analysis; final approval of the manuscript; study design; implemen-tation of projects and/or experiments; manuscript writing or critical review of its content
